# Predictors of preeclampsia with severe features: A multi–center analysis of maternal and health system factors in Thai tertiary hospitals

**DOI:** 10.1371/journal.pone.0361195

**Published:** 2026-10-21

**Authors:** Tippawan Boonyaporn, Piyanun Limruangrong, Ameporn Ratinthorn, Thitipong Tankumpuan

**Affiliations:** 1 Doctor of Nursing Science (Candidate), Faculty of Nursing, Mahidol University, Bangkok, Thailand; 2 Department of Obstetrics and Gynaecology Nursing, Faculty of Nursing, Mahidol University, Bangkok, Thailand; 3 Department of Surgical Nursing, Faculty of Nursing, Mahidol University, Bangkok, Thailand; University of Nebraska Medical Center College of Medicine, UNITED STATES OF AMERICA

## Abstract

**Background:**

Preeclampsia (PE) with severe features threatens maternal and neonatal health. This study examined maternal and health–service factors associated with severe features and compared maternal and neonatal outcomes in Thailand.

**Methods:**

This case–control study enrolled 156 postpartum women with PE (52 with and 104 without severe features) and 111 nurse–midwives in antenatal care, labor, and postpartum units across four Thai tertiary hospitals. Data were collected using postpartum and nurse–midwife questionnaires, and a maternal–neonatal health record form. Group differences were assessed using independent t–test, chi–square or Fisher’s exact tests. Multivariable binary logistic regression examined factors associated with severe features, adjusting for antenatal care visits and gravidity.

**Results:**

Women with severe features more often had a history of PE (32.7% vs. 13.5%, p = 0.006) and differed in BMI distribution (p = 0.007). Three maternal factors were independently associated with severe features: history of PE (aOR = 5.99, 95% CI 2.13–16.83), BMI (aOR = 0.92 per kg/m^2^, 95% CI 0.86–0.98), and moderate versus low PE knowledge (aOR = 0.13, 95% CI 0.04–0.50). No health–service variable, including nurse training, was independently associated with severe features after adjustment (all p > 0.05). The model explained 29.4% (Nagelkerke R^2^) of the variance in PE severity and correctly classified 75.8% of cases. Infant birth weight was lower among women with severe features (p < 0.05); delivery mode, hospital stay, birth asphyxia, and NICU admission did not differ significantly.

**Conclusion:**

A history of PE was associated with higher odds, and moderate (versus low) PE knowledge and higher BMI with lower odds, of severe features. These findings support prioritizing screening for prior PE history and structured antenatal knowledge counseling, together with adequately powered, multilevel studies to clarify the role of health–service factors in PE severity.

## Introduction

Preeclampsia (PE) is a significant complication of pregnancy, presenting substantial health risks to both mothers and newborns. A recent meta–analysis estimated a global PE prevalence of 4.43% [1]. Hypertensive disorders of pregnancy, including PE, accounted for an estimated 16% of maternal deaths worldwide during 2009–2020 [2]. Reported PE rates varied between two Thai tertiary hospitals: 1.9% of deliveries during 2012–2016 [3] and 9.1% during a three–month period in 2023 [4].

The consequences of PE are particularly pronounced when severe features develop. In the earlier Thai study, postpartum hemorrhage occurred in 9.9% of women with PE. Compared with nonsevere PE, severe features or HELLP (hemolysis, elevated liver enzymes, and low platelet count) syndrome were associated with more low–birth–weight infants and neonatal intensive care unit (NICU) admissions [3]. A separate case–control study found higher cesarean delivery rates and longer hospital stays among women with severe PE than among women without severe PE [5]. A history of PE has also been associated with later maternal cardiovascular disease [6], while a US study documented substantial healthcare costs for mothers and infants [7].

Caring for pregnant women with PE requires attention to maternal characteristics and the care they receive. This study adapted the Donabedian structure–process–outcome model [8] and the World Health Organization’s framework for maternal and newborn care quality [9] to organize maternal and health–service inputs, care processes, and outcomes. Maternal age ≥ 35 years, previous PE, body mass index (BMI) ≥25 kg/m^2^, and comorbidities such as chronic hypertension and pregestational diabetes have been associated with PE with severe features when affected women were compared with a broader obstetric population in a Thai hospital [10]. However, these findings do not necessarily identify factors that distinguish severe from nonsevere PE among women who already have PE. An earlier Thai study that directly compared these two PE groups found that several maternal characteristics, including previous PE and underlying medical conditions, did not differ significantly [3].

Maternal knowledge, calcium intake, and exercise were also considered. In an Ethiopian study, 28.8% of pregnant women had good knowledge of PE [11]. The WHO recommends calcium supplementation during pregnancy in populations with low dietary calcium intake to reduce PE risk [12], and a meta–analysis found lower odds of developing PE with prenatal exercise interventions [13]. However, these studies did not establish whether these factors distinguish severe from nonsevere PE among women already diagnosed with the condition.

Access to care presents another possible influence. In Haiti, women with PE experienced longer delays in reaching emergency obstetric care than women without PE (14.6 vs. 6.8 hours) [14]. Research in Zambia and Uganda found that transport modes and travel times for obstetric care differed by household wealth; women also reported difficulties related to cost, distance, and transport availability [15]. These studies support examining distance and transportation costs, but they do not establish that either factor increases PE severity in Thailand. Evidence on the combined role of maternal and access–related factors among Thai women with PE remains limited.

Health–service inputs and care processes may also affect the care women receive. Studies in Nigeria have assessed providers’ PE knowledge and the quality of antenatal care for hypertensive disorders of pregnancy [16,17]. Evidence on midwife experience is mixed: one New Zealand cohort found higher perinatal mortality among women cared for by midwives in their first year of practice [18], whereas a later cohort did not confirm an increased risk after adjustment [19]. The International Federation of Gynecology and Obstetrics (FIGO) has emphasized matching maternity staffing to delivery volume, case mix, and required skills [20]. Emergency obstetric care training has improved providers’ assessed knowledge and skills, including those related to PE [21]. These findings justify examining nurses’ experience, staffing, training, knowledge, and care processes, without assuming that they independently predict PE severity.

Thailand’s Second National Reproductive Health Development Policy and Strategy (2017–2026) emphasizes safe motherhood, quality maternal and child health services, and equitable access to care [22]. Nevertheless, few studies have examined maternal and health–service factors together among Thai women already diagnosed with PE. This case–control study therefore examined associations between these factors and severe features and compared maternal and neonatal outcomes between women with and without severe features.

## Methods

### Design and study sites

This study employed an analytical case–control design. The case–control approach was selected to investigate associations between specific exposures (maternal and health–system factors) and the outcome (preeclampsia with severe features), an approach well suited to studying this relatively uncommon pregnancy complication. Data collection followed a cross–sectional timeline at participating tertiary hospitals in Thailand. According to the Ministry of Public Health, tertiary hospitals are recognized as medical facilities with the capacity and preparedness to manage preeclamptic women. Four tertiary hospitals, randomly selected from 33 centers nationwide, were chosen as research sites through multi–stage random selection to enhance representativeness. The study included one hospital from each region of Thailand, all adhering to the same Clinical Practice Guidelines (CPG) for managing preeclampsia.

### Population and samples

This case–control study included postpartum women with physician–diagnosed preeclampsia and nurse–midwives at four Thai tertiary hospitals. Cases were women with preeclampsia with severe features, and controls were women with preeclampsia without severe features. Women were eligible if they were aged 18 years or older, had a singleton pregnancy, initiated antenatal care (ANC) before 28 weeks of gestation, attended ANC for at least three months, and could communicate in Thai. Women with a diagnosed psychiatric disorder, chronic hypertension, or cardiac disease were excluded.

The final analytic sample comprised 156 postpartum women: 52 cases with severe features and 104 controls without severe features, an observed case–to–control ratio of 1:2. The methods used a formula for comparing two independent proportions, as described by Kelsey et al. [23], to plan the case–control sample size. The recorded inputs were ($P_{\text{1}}$=0.5217), ($P_{\text{2}}$=0.2943) [24], a two–sided significance level of 0.05, and a target power of 0.85:


$$\begin{array}{c} n_{cases}\;=\;\frac{r\;+\;\text{1}}{r}\;\times \;\frac{p¯(\text{1}\;-\;p¯){\left(Z_{\text{1}-\alpha /\text{2}}\;+\;Z_{\text{1}-\beta }\right)}^{\text{2}}}{{\left(P_{\text{1}}\;-\;P_{\text{2}}\right)}^{\text{2}}},\text{\textasciitilde{}}p¯\;=\;\frac{P_{\text{1}}\;+\;rP_{\text{2}}}{r\;+\;\text{1}} \end{array}$$

The sampling frame comprised 150 eligible nurses and midwives working in antenatal care, labor and delivery, and postpartum units across the four hospitals. For a population of 150, the Krejcie and Morgan table lists a sample size of 108, based on a 95% confidence level, an assumed population proportion of 0.50, and a margin of error of 0.05 [25]. The target sample was set at 111 nurses and midwives. Following proportional allocation across hospitals and units, participants were selected by simple random sampling. The final sample comprised 111 nurses and midwives (74.0% of the sampling frame).

### Research instrument

The study utilized three data collection instruments, developed through a review of the literature [11,16,17,26] and guidelines from the Royal Thai College of Obstetricians and Gynaecologists [27], the American College of Obstetricians and Gynecologists [28], and the National Institute for Health and Care Excellence [29]: (1) the postpartum women’s questionnaire, (2) the nurse–midwife questionnaire, and (3) the maternal–neonatal health record form. The postpartum women’s questionnaire comprised three domains: demographic characteristics, preeclampsia knowledge assessment, and a maternity care service utilization questionnaire. The knowledge assessment contained 30 dichotomous (true/false) items, with higher scores denoting greater knowledge. Total knowledge scores ranged from 0–30 and were categorized, following Bloom’s cut–off convention [30], as low (0–18), moderate (19–24), or high (25–30). The maternity care service questionnaire incorporated structured and open–ended items, with cumulative scores reflecting utilization intensity. Composite care–process scores ranged from 0–42 and were divided into three equal intervals: low (0–14), moderate (15–28), and high (29–42) levels of care received.

The nurse–midwife questionnaire evaluated practitioners’ preeclampsia–related knowledge, management competencies, and counseling capabilities, encompassing demographic characteristics and knowledge/skills assessment through multiple–choice items; knowledge categorization paralleled the methodology used in the postpartum questionnaire, applying identical thresholds.

The maternal–neonatal health record form comprised 21 clinical parameters documenting maternal and infant characteristics, including gestational age, body mass index (BMI), peripartum complications, and duration of hospitalization. Content validity was assessed by three experts (two physicians and one advanced practice nurse), yielding a scale–content validity index (S–CVI) exceeding 0.8 for all instruments. Reliability was assessed through pilot testing with 30 postpartum women and 10 nurse–midwives, yielding Cronbach’s alpha coefficients of 0.709 for both the postpartum women’s and nurse–midwives’ questionnaires, indicating an acceptable, though modest, level of internal consistency.

### Ethical considerations

This research project was approved by the Institutional Review Board of the Faculty of Nursing, Mahidol University (COA No. IRB–NS2023/740.1801; approval date: 18 January 2023). Ethical approval was subsequently obtained from the ethics committees of three participating tertiary hospitals: Chiangrai Prachanukroh Hospital (Approval No. EC CRH 025/66 Ex; approval date: 9 May 2023), Sapphasitthiprasong Hospital (Approval No. 085/65 S; approval date: 29 December 2022), and Ratchaburi Hospital (Approval No. 009/66; approval date: 29 January 2023). As Krabi Hospital does not maintain its own ethics committee, approval from the Institutional Review Board of the Faculty of Nursing, Mahidol University was applied to this study site.

The study was conducted in accordance with the Declaration of Helsinki. Participant recruitment occurred at Chiangrai Prachanukroh Hospital from 15 May to 15 October 2023, Sapphasitthiprasong Hospital from 15 January to 30 June 2023, Ratchaburi Hospital from 1 February to 31 July 2023, and Krabi Hospital from 20 January to 20 August 2023. Written informed consent was obtained from all participants (postpartum women and nurses) using approved consent forms; all participants were informed of their right to withdraw at any time without penalty, and the requirement for informed consent was not waived by any of the ethics committees.

### Methods of data collection

The data collection protocol adhered to rigorous ethical and methodological standards. Following approval from the Institutional Review Board of the Faculty of Nursing, Mahidol University and the Institutional Review Boards of the four participating hospitals (Chiangrai Prachanukroh, Sapphasitthiprasong, Ratchaburi, and Krabi Hospitals), formal communication was sent from the Graduate Studies Office to secure institutional access. Initial consultations with key stakeholders—heads of obstetrics and gynecology departments, postpartum unit supervisors, and nursing administrators—were conducted to outline the research objectives and procedures.

Research assistants were recruited from registered nurses in postpartum units, with selection criteria requiring a minimum of five years of midwifery experience. These assistants underwent comprehensive training in data collection protocols, with emphasis on participant rights protection and confidentiality. The data collection process began with identification of eligible participants, followed by presentation of study objectives and procurement of informed consent. Self–administered questionnaires, requiring approximately 45–60 minutes to complete, were distributed, with the research team providing clarification for ambiguous or incomplete responses. Maternal and neonatal health data were concurrently extracted from medical records.

Data collection progress was monitored biweekly to ensure adequate response rates and case acquisition. Healthcare providers, including nurses–midwives and departmental heads from antenatal care, labor, and postpartum units, were recruited through a parallel process; after informed consent, providers completed a questionnaire requiring 10–20 minutes. Incomplete datasets were excluded from analysis to ensure data integrity while maintaining participant confidentiality.

### Data analysis

Data were analyzed using IBM SPSS Statistics for Windows, Version 22.0. Data cleaning was performed to ensure completeness and validity prior to analysis. Descriptive statistics, including frequency, percentage, mean, and standard deviation, were used to summarize postpartum data and healthcare service characteristics. The chi–square test or Fisher’s exact test (for cells with expected counts <5) assessed differences in proportions between groups, at a significance level of 0.05.

Candidate predictors for the multivariable model were identified a priori from the conceptual framework (Donabedian input–process structure [8] and the WHO maternal care quality framework [9]) and from the literature reviewed in the Introduction, rather than being restricted to variables that were statistically significant on univariate analysis; this approach was adopted to preserve theoretically and clinically relevant predictors that might otherwise be omitted due to limited statistical power at the univariate level. All 14 candidate predictors were entered simultaneously using multivariable binary logistic regression (forced–entry method) to identify factors independently associated with preeclampsia with severe features, given the dichotomous nature of the outcome (severe versus non–severe features). The final adjusted model additionally controlled for two covariates: number of ANC visits (entered as a continuous variable, range 4–14 visits) and gravidity (entered as a continuous variable, range 1–6 pregnancies), selected on the basis of their established clinical relevance as confounders of preeclampsia severity. Model fit was evaluated using the overall likelihood–ratio chi–square test, and the proportion of variance explained was reported using Cox & Snell and Nagelkerke pseudo–R^2^ statistics; classification accuracy was assessed against the observed outcome. Multicollinearity among predictors was assessed using variance inflation factors (VIF), with all values below 2.5, indicating no problematic collinearity; model calibration was evaluated using the Hosmer–Lemeshow goodness–of–fit test (p > 0.05, indicating adequate fit). Adjusted odds ratios (aOR) with 95% confidence intervals are reported for all predictors, and statistical significance was defined as p < 0.05, denoted with a single asterisk (*) in Table 1,3, and 4; p < 0.01 is denoted with two asterisks (**), Open–ended responses were summarized qualitatively to support interpretation of the quantitative findings.

## Results

### Sample characteristics and univariate comparisons

Of 208 postpartum women with preeclampsia screened across the four participating hospitals, 156 met all eligibility criteria and were included in the final analysis (52 cases with severe features, 104 controls without severe features), consistent with the *a priori* sample size requirement and the denominator used throughout this manuscript. This study compared postpartum women with and without severe features of preeclampsia, revealing statistically significant between–group differences in history of preeclampsia, monthly meal expenses, and body mass index (BMI) (Table 1). Among women with severe features, 44.2% were aged ≥35 years and 32.7% had a history of preeclampsia in a previous pregnancy, compared with 10.6% and 13.5%, respectively, among controls (history of preeclampsia: p = 0.006). BMI distribution also differed significantly between groups (p = 0.007): women with severe features were more concentrated in the overweight category (BMI 25.0–29.9 kg/m^2^: 55.8% vs. 33.7%), whereas a higher proportion of controls fell in the obese category (BMI ≥ 30 kg/m^2^: 36.5% vs. 15.4%), indicating a non–monotonic distribution that is examined further in the multivariable analysis below. Maternal age, comorbidity status, and exercise habits did not differ significantly between groups (all p > 0.05).

**Table 1:** Frequency and percentage of maternal factors among postpartum women with preeclampsia, by severity status (N = 156).

Maternal factor	Severe features (n = 52) n (%)	Without severe features (n = 104) n (%)	Total (N = 156) %	p–value
Age < 20 years	4 (7.7)	8 (7.7)	**7.7**	0.313
Age 20–34 years	25 (48.1)	85 (81.7)	**70.5**	
Age ≥ 35 years	23 (44.2)	11 (10.6)	**21.8**	
History of PE – No	35 (67.3)	90 (86.5)	80.1	0.006*
History of PE – Yes	17 (32.7)	14 (13.5)	19.9	
Comorbidity – No	35 (67.3)	72 (69.2)	68.6	0.856
Comorbidity – Yes	17 (32.7)	32 (30.8)	31.4	
Meal expenses ≤100 baht	17 (32.7)	53 (51.0)	44.9	<0.001*
Meal expenses 101–200 baht	10 (19.2)	10 (9.6)	12.8	
Meal expenses ≥201 baht	25 (48.1)	41 (39.4)	42.3	
BMI < 25.0 kg/m²	15 (28.8)	31 (29.8)	29.5	0.007*
BMI 25.0–29.9 kg/m^2^	29 (55.8)	35 (33.7)	41.0	
BMI ≥ 30 kg/m^2^	8 (15.4)	38 (36.5)	29.5	
Calcium intake – Yes	45 (86.5)	95 (91.3)	89.7	0.264^f^
Calcium intake – No	7 (13.5)	9 (8.7)	10.3	
Knowledge – Low (0–18)	9 (17.3)	5 (4.8)	9.0	0.257
Knowledge –Moderate (19–24)	8 (15.4)	32 (30.8)	25.6	
Knowledge – High (25–30)	35 (67.3)	67 (64.4)	65.4	

^f^Fisher’s exact test. *Significant at p < 0.05.

### Nurse–midwife characteristics and care process

Most nurse–midwives had more than ten years of work experience (mean = 12.68 years, SD = 9.78). The majority had not received specific training on preeclampsia, although expertise was highest in exercise counseling for expectant mothers, followed by preeclampsia screening and weight–control counseling. More than half of nurse–midwives demonstrated a moderate level of knowledge about preeclampsia and generally maintained appropriate nurse–to–patient ratios.

The care process for postpartum women with and without severe features of preeclampsia was compared (Table 2). No statistically significant difference was found between groups (p = 0.551); however, a markedly different distribution pattern was observed, with women who had severe features more frequently receiving a moderate level of overall care (75.0% vs. 32.7%), whereas women without severe features more frequently received a high level of care (64.4% vs. 25.0%).

**Table 2:** Frequency and percentage of care–process levels among postpartum women with preeclampsia, by severity status (N = 156).

Care process level	Severe features (n = 52) n (%)	Without severe features (n = 104) n (%)	Total (N = 156) %	p–value
Low (0–14)	0 (0.0)	3 (2.9)	1.9	0.551
Moderate (15–28)	39 (75.0)	34 (32.7)	46.8	
High (29–42)	13 (25.0)	67 (64.4)	51.3	
Total	52 (100.0)	104 (100.0)	100.0	

Mean composite care–process score = 27.22 (SD 8.403; range 8–42).

### Maternal and neonatal outcomes

Comparative maternal and neonatal outcomes between women with and without severe features of preeclampsia are summarized in Table 3. Infant birth weight was significantly lower among women with severe features compared with controls (p = 0.006). No statistically significant between–group differences were observed for mode of delivery, length of hospital stay, birth asphyxia, or NICU admission (all p > 0.05), consistent with the qualitative pattern described in the Discussion.

**Table 3:** Maternal and neonatal outcomes by preeclampsia severity status (N = 156).

Outcome	Severe features (n = 52)	Without severe features (n = 104)	p–value
Infant birth weight, < 2,500 g	23 (44.2)	33 (31.7)	0.006*
Infant birth weight, 2,501–2,999 g	11 (21.2)	38 (36.5)	
Infant birth weight, 3,000–3,499 g	13 (25)	25 (24.0)	
Infant birth weight, 3,500–3,999 g	5 (9.6)	7 (6.7)	
Infant birth weight, > 4,000 g	0 (0.0)	1 (1.0)	
Mode of delivery – Normal delivery	11(21.2)	17(16.3)	0.082
Mode of delivery – Vacuum extraction	0(0.0)	9(8.7)	
Mode of delivery – caesarean delivery	41(78.8)	78(75.0)	
Length of hospital stay, 0–3 days	42 (80.8)	94 (90.4)	0.333
Length of hospital stay, 4–7 days	8 (15.4)	10 (9.6)	
Length of hospital stay, > 7 days	2 (3.8)	0 (0.0)	
Birth asphyxia –Yes	6 (11.5)	15 (14.4)	0.804^f^
Birth asphyxia –No	46(88.5)	89(85.6)	
NICU admission–Yes	15 (28.8)	22 (21.2)	0.456^f^
NICU admission–No	37 (71.2)	82 (78.8)	

^f^Fisher’s exact test (expected cell count <5). *Significant at p < 0.05.

### Multivariable analysis of input and process factors

Multivariable binary logistic regression was performed to identify factors independently associated with preeclampsia with severe features (Table 4). The full model, containing 14 predictors entered simultaneously and adjusted for number of ANC visits and gravidity, was statistically significant, χ^2^(14) = 36.74, p < 0.001, and correctly classified 75.8% of cases. The model explained 21.2% (Cox & Snell R^2^) to 29.4% (Nagelkerke R^2^) of the variance in preeclampsia severity. Model diagnostics indicated an adequate fit (Hosmer–Lemeshow test, χ**^2^** = 6.14, df = 8, p = 0.631 > 0.05) and no problematic multicollinearity among predictors (variance inflation factors ranged from 1.05 to 1.87, all below the threshold of 2.5).

**Table 4:** Crude and adjusted odds ratios for input and process factors associated with preeclampsia with severe features (N = 156).

Factor	Crude OR	95% CI	p–value	Adjusted OR	95% CI	p–value
**Maternal factors**						
Age	0.972	0.911–1.036	0.383	1.044	0.985–1.106	0.147
BMI	0.912	0.843–0.989	0.025*	0.917	0.857–0.982	0.013*
History of PE	7.018	1.995–15.263	0.002**	5.985	2.129–16.825	0.001**
Calcium	0.974	0.202–4.102	0.902	1.762	0.593–5.241	0.308
Comorbidity	0.485	0.182–1.480	0.220	1.182	0.556–2.514	0.664
Distance	1.011	0.996–1.026	0.138	1.004	0.993–1.016	0.458
Exercise	0.863	0.359–2.075	0.528	0.842	0.423–1.678	0.626
Transportation cost	1.000	1.000–1.000	0.634	1.000	1.000–1.000	0.354
Knowledge – Low	1 (ref)	—	—	1 (ref)	—	—
Knowledge – Moderate	0.128	0.023–0.617	0.011*	0.134	0.036–0.502	0.003**
Knowledge – High	0.392	0.089–1.639	0.195	0.346	0.105–1.136	0.080
**Health–service system factors**						
Nurse training	2.416	0.920–6.720	0.073	1.891	0.927–3.856	0.080
Nurse–to–patient ratio	0.556	0.224–1.384	0.208	0.570	0.271–1.197	0.138
Nursing experience	0.394	0.112–1.260	0.113	0.990	0.786–1.248	0.934
Nurses’ knowledge	0.026	0.000–1.348	0.070	1.004	0.478–2.109	0.992
**Process factor**						
Care–process score	1.020	0.976–1.057	0.458	1.017	0.977–1.059	0.414

Model: −2 Log likelihood = 160.22; χ^2^(14) = 36.74, p < 0.001; Cox & Snell R^2^ = 0.212; Nagelkerke R^2^ = 0.294; classification accuracy = 75.8%; Hosmer–Lemeshow χ^2^(8) = 6.14, p = 0.631; all VIF < 1.87. Adjusted for number of ANC visits and gravidity. *p < 0.05, **p < 0.01.

Three variables made statistically significant, independent contributions to the model. First, a history of preeclampsia was associated with approximately sixfold higher odds of severe features (aOR = 5.99, 95% CI 2.13–16.83, p = 0.001). Second, BMI was associated with modestly lower odds of severe features, with each 1 kg/m^2^ increase corresponding to an 8% reduction in adjusted odds (aOR = 0.92, 95% CI 0.86–0.98, p = 0.013); this direction is consistent with the non–monotonic distribution observed in Table 1 and is examined further in the Discussion. Third, compared with women with low knowledge scores, those with moderate knowledge scores had substantially lower adjusted odds of severe features (aOR = 0.13, 95% CI 0.04–0.50, p = 0.003); the high–knowledge category did not reach statistical significance relative to the low–knowledge reference group (aOR = 0.35, 95% CI 0.11–1.14, p = 0.080).

The remaining 11 variables were not statistically significant independent predictors in the adjusted model (all p > 0.05), including maternal age, comorbidity, calcium supplementation, exercise, distance to hospital, transportation cost, nurse training, nurse–to–patient ratio, nursing experience, nurses’ knowledge, and care–process score. Nurse training showed a trend toward association that did not reach significance (aOR = 1.89, 95% CI 0.93–3.86, p = 0.080) and should not be interpreted as an independent predictor in this sample.

## Discussion

This study examined maternal and health–system factors associated with the severity of preeclampsia among women already diagnosed with the condition. A history of preeclampsia in a previous pregnancy was the strongest independent predictor of severe features (aOR = 5.99, 95% CI 2.13–16.83), consistent with evidence that preeclampsia is associated with persistent vascular and endothelial changes [31,32] and with a substantial risk of recurrence in subsequent pregnancies [33]. In contrast, BMI showed an inverse association with severity in the adjusted model (aOR = 0.92 per kg/m^2^, 95% CI 0.86–0.98): each unit increase in BMI corresponded to a modest reduction, rather than an increase, in the odds of severe features. This finding departs from the conventional epidemiological association between elevated BMI and preeclampsia risk reported in general obstetric populations [34,35]. A plausible explanation lies in the case–control design itself: because both groups in this study already had a diagnosis of preeclampsia, the comparison reflects factors distinguishing severity within an already–affected population, rather than the determinants of disease onset captured by these studies. Consistent with this, an earlier Thai study comparing women with and without severe features found lower pre–pregnancy BMI among those with severe features [3]. This distinction is consistent with the non–monotonic BMI distribution observed in Table 1, where the highest proportion of severe cases occurred in the overweight category (25.0–29.9 kg/m^2^) rather than the obese category (≥30 kg/m^2^), suggesting that the relationship between BMI and severity may not be linear across the full BMI range. Given the modest case sample size (n = 52), this estimate should be interpreted cautiously, and future studies with categorical or spline modeling of BMI are warranted to clarify this pattern.

Maternal knowledge of preeclampsia did not differ significantly between–group in the univariate analysis (Table 1, p = 0.257). However, after adjustment, it emerged as a significant independent predictor, women with moderate knowledge had substantially lower odds of severe features than those with low knowledge (aOR = 0.13, 95% CI 0.04–0.50, p = 0.003). This finding is consistent with a potential suppression effect, whereby the crude association between maternal knowledge and disease severity may have been obscured by its relationships with a history of preeclampsia and BMI. After adjustment for these variables, the inverse association became evident. This interpretation is consistent aligns with prior evidence indicating that limited awareness of warning signs may delays care–seeking and contribute to poorer clinical outcomes [36]. These findings highlight maternal health literacy as a potentially modifiable target for antenatal counseling.

Care–process levels did not differ significantly between groups overall (p = 0.551); however, women with severe features more often received only moderate–level care (75.0%) while controls more often received high–level care (64.4%), a pattern that, although not reaching the conventional significance threshold, is directionally consistent with international guidance emphasizing intensive monitoring and prompt antihypertensive management for high–severity cases [27,37]. This may explain why infant birth weight, an outcome sensitive to placental and vascular compromise, differed significantly between groups (Table 3) [38] even though the composite process–of–care score did not: broad process indicators may be too coarse to capture the specific, time–critical interventions that influence perinatal outcomes.

Health–system variables—nurse training, nurse–to–patient ratio, nursing experience, and nurses’ knowledge—were not independently associated with the presence of severe features, despite their theoretical importance [16,18–21,39]. Several explanations merit consideration. First, statistical power to detect associations involving these provider–level variables may have been limited by the small number of cases (n = 52). Second, because all four participating hospitals operated under the same national Clinical Practice Guidelines, between–hospital variability in provider practice may have been limited, reducing the observed effect. Third, nurse–level variables were linked to patient–level outcomes without accounting for clustering of patients and nurses within hospitals, which may have attenuated true associations; multilevel modeling in future studies with larger provider samples would help clarify this relationship. Nurse training was associated with higher odds of severe features, although the association did not reach statistical significance (aOR = 1.89, p = 0.080). Therefore, this finding should be interpreted cautiously and should not support an independent association in the present sample.

Collectively, these findings indicate that maternal history and knowledge—rather than health–system process indicators—were the dominant correlates of severity within this cohort of women already diagnosed with preeclampsia, highlighting maternal risk stratification and targeted education as priority areas for clinical attention, while cautioning against overstating the role of BMI or provider–level factors given the direction and precision of the estimates obtained.

### Strengths and limitations

This study demonstrates several strengths, including a well–defined research objective and a rigorous methodology incorporating a case–control design with multi–stage random selection of tertiary hospitals across Thailand. The use of the Donabedian model and WHO framework provides a structured approach to analyzing healthcare services for women with preeclampsia, and the study effectively contextualizes its results by referencing relevant international literature. The a priori calculation of sample size, together with model diagnostics confirming adequate fit and the absence of problematic multicollinearity, further support the statistical reliability of the multivariable findings.

Several limitations should nonetheless be acknowledged. First, the study focused exclusively on tertiary hospitals, which limits generalizability to lower–level healthcare facilities and to community–based antenatal care settings. Second, because both the case and control groups comprised women already diagnosed with preeclampsia, the findings describe correlates of disease severity rather than determinants of disease onset. Accordingly, the observed inverse association between BMI and severity should not be generalized to the general obstetric population. Third, the internal consistency of the study instruments was modest (Cronbach’s alpha = 0.709 for both the postpartum women’s and nurse–midwives’ questionnaires), at the lower boundary of conventional acceptability, which may have reduced the precision of associations involving the knowledge and care–process scores. Fourth, the health–system variables (nurse training, staffing ratio, experience, and knowledge) were analyzed at the individual nurse level without accounting for clustering of nurses and patients within the four hospitals. This hierarchical structure may have affected the precision of the estimates and should be addressed in future studies using multilevel modeling. Fifth, the small number of severe cases (n = 52) limited the statistical power to detect modest associations with health–system predictors and the ability to examine potential nonlinearity in the association between BMI and disease severity. Finally, self–reported data on knowledge, exercise, and healthcare expenditure may be susceptible to recall and social desirability bias, while the cross–sectional design precludes causal inference regarding the temporal sequence between exposures and disease severity.

## Conclusion

This study examined maternal and health–system factors associated with the severity of preeclampsia among women already diagnosed with the condition, comparing those with and without severe features. A history of preeclampsia in a previous pregnancy and maternal knowledge of preeclampsia were independently associated with severity: women with a prior history had approximately sixfold higher adjusted odds of severe features (aOR = 5.99), while moderate knowledge, relative to low knowledge, was associated with markedly lower odds (aOR = 0.13). BMI showed a modest inverse association with severity in the adjusted model (aOR = 0.92 per kg/m^2^), a finding that should be interpreted within the case–control comparison of already–affected women rather than as evidence that higher BMI protects against preeclampsia in general. Health–system variables—including nurse training, staffing ratios, nursing experience, and overall care–process scores—were not independently associated with severity after adjustment, although nurse training showed a non–significant trend that warrants further investigation in adequately powered, multilevel study designs. In addition, infant birth weight was significantly lower among women with severe features, underscoring the clinical relevance of maternal history and knowledge–based risk stratification for perinatal outcomes.

These findings support prioritizing systematic screening for prior preeclampsia history and structured antenatal knowledge counseling as practical, low–cost strategies to identify women at elevated risk of severe disease. Given the limitations of statistical power and the single–level analytic approach applied to health–system variables, further multi–center studies with larger provider samples and multilevel modeling are needed before firm conclusions can be drawn regarding the independent contribution of nurse training and staffing to preeclampsia severity in Thai tertiary hospitals.
